# Neuromodulation of Hippocampal-Prefrontal Cortical Synaptic Plasticity and Functional Connectivity: Implications for Neuropsychiatric Disorders

**DOI:** 10.3389/fncel.2021.732360

**Published:** 2021-10-11

**Authors:** Rafael Naime Ruggiero, Matheus Teixeira Rossignoli, Danilo Benette Marques, Bruno Monteiro de Sousa, Rodrigo Neves Romcy-Pereira, Cleiton Lopes-Aguiar, João Pereira Leite

**Affiliations:** ^1^Department of Neuroscience and Behavioral Sciences, Ribeirão Preto Medical School, University of São Paulo, Ribeirão Preto, Brazil; ^2^Department of Physiology and Biophysics, Institute of Biological Sciences, Federal University of Minas Gerais, Belo Horizonte, Brazil; ^3^Brain Institute, Federal University of Rio Grande do Norte, Natal, Brazil

**Keywords:** hippocampus, prefrontal cortex, long-term potentiation (LTP), long-term depression (LTD), local field potential (LFP), acetylcholine, monoamines, cannabinoids

## Abstract

The hippocampus-prefrontal cortex (HPC-PFC) pathway plays a fundamental role in executive and emotional functions. Neurophysiological studies have begun to unveil the dynamics of HPC-PFC interaction in both immediate demands and long-term adaptations. Disruptions in HPC-PFC functional connectivity can contribute to neuropsychiatric symptoms observed in mental illnesses and neurological conditions, such as schizophrenia, depression, anxiety disorders, and Alzheimer’s disease. Given the role in functional and dysfunctional physiology, it is crucial to understand the mechanisms that modulate the dynamics of HPC-PFC communication. Two of the main mechanisms that regulate HPC-PFC interactions are synaptic plasticity and modulatory neurotransmission. Synaptic plasticity can be investigated inducing long-term potentiation or long-term depression, while spontaneous functional connectivity can be inferred by statistical dependencies between the local field potentials of both regions. In turn, several neurotransmitters, such as acetylcholine, dopamine, serotonin, noradrenaline, and endocannabinoids, can regulate the fine-tuning of HPC-PFC connectivity. Despite experimental evidence, the effects of neuromodulation on HPC-PFC neuronal dynamics from cellular to behavioral levels are not fully understood. The current literature lacks a review that focuses on the main neurotransmitter interactions with HPC-PFC activity. Here we reviewed studies showing the effects of the main neurotransmitter systems in long- and short-term HPC-PFC synaptic plasticity. We also looked for the neuromodulatory effects on HPC-PFC oscillatory coordination. Finally, we review the implications of HPC-PFC disruption in synaptic plasticity and functional connectivity on cognition and neuropsychiatric disorders. The comprehensive overview of these impairments could help better understand the role of neuromodulation in HPC-PFC communication and generate insights into the etiology and physiopathology of clinical conditions.

## Introduction

A remarkable diversity of neural circuits in the mammalian brain provides a substrate for adaptive and maladaptive behavioral responses (Deisseroth, 2014). The HPC-PFC circuit plays a fundamental role in cognitive functions, such as short-term and long-term memory, attention, and decision-making, which are affected by several neurological diseases and psychiatric disorders (Kovner et al., 2019). In rodents, the anatomical description of the hippocampal-prefrontal pathway has been combined with long-range and local electrophysiological measures to investigate the processing of neural information (Laroche et al., 2000; Takita et al., 2013). Noteworthy, the adjustment and perpetuation of the information in the hippocampal-prefrontal pathway occur through short-term plasticity and long-term plasticity, respectively (Citri and Malenka, 2008). The HPC-PFC pathway is neuromodulated by several neurotransmitter systems: cholinergic, monoaminergic, and endocannabinoid (Goto et al., 2010; Puig and Gener, 2015; Ruggiero et al., 2017; Ranjbar-Slamloo and Fazlali, 2020). The neuromodulation of the HPC-PFC pathway is essential to a fine-tune regulation of the circuit, affecting the synaptic efficacy, synaptic plasticity, and oscillatory patterns implicated in behavioral and circuit alterations related to neuropsychiatric conditions (Godsil et al., 2013).

This review outlines the neuromodulation of the synaptic plasticity and network coupling in the hippocampal-prefrontal pathway underlying relevant aspects of the neuroanatomy and electrophysiological measures in rodents. We also examine the disruption of these circuits related to mal-adaptive impairments and provide a critical discussion for new potential developments in the field.

## Hippocampus-Prefrontal Cortex Anatomical Projection

The hippocampus is probably one of the most studied brain regions and is well known to exert a critical role in semantic memory formation and spatial learning. The hippocampal formation is localized in the temporal lobe and is constituted by the *Cornu Ammonis* (CA) fields (CA1, CA2, and CA3), the dentate gyrus, the subicular complex, and the entorhinal cortex (Andersen et al., 2009). In rodents, the hippocampal formation extends in a C-shaped manner through a dorsal (septal) to a ventral (temporal) axis, corresponding to the posterior to the anterior axis in humans (Andersen et al., 2009; Strange et al., 2014). Lesion and connectivity studies indicate a functional distinction in the hippocampus, with the dorsal part mediating cognitive aspects (especially spatial memory) and the ventral region modulating emotional processes (Groenewegen et al., 1987; Van Groen and Wyss, 1990; Moser et al., 1993; Risold and Swanson, 1996). More recently, genetic expression domain studies have shown a tripartite profile that divides the dentate gyrus and CA1 into dorsal, intermediate, and ventral portions (Lein et al., 2007). Furthermore, extrinsic connectivity shows a topographical representation - both from the neocortex to the hippocampus and from the hippocampus to subcortical structures - presenting a gradual transition along the septo-temporal axis (Strange et al., 2014).

The PFC is a brain region that presents marked differences during phylogenetic development. Compared to other mammal species, the PFC of primates shows a dramatic volume increase and differentiation (Fuster, 2008). Several authors discuss if the murine species used in neuroscience research present a prefrontal cortex (Carlén, 2017; Laubach et al., 2018). The classical definition of the PFC (Rose and Woolsey, 1948) is the cortical projection area of the mediodorsal thalamic nucleus. This broad definition included all mammals as possessing a frontal region equivalent to the primate frontal granular cortex (Carlén, 2017) and motivated initial studies of functional similarity between the PFC of rodents and the higher cognitive dorsolateral prefrontal cortex (dlPFC) of primates (Laubach et al., 2018). However, more recent literature indicates notable differences in the functional aspects of the PFC and the dlPFC of humans (see Carlén, 2017; Laubach et al., 2018 for a review). Indeed, the granular dorsolateral PFC is considered to be unique to primates (Wise, 2008), and cytoarchitecture evidence indicates that the rodent PFC is homologous to the human anterior cingulate cortex (ACC) (Vogt et al., 2013; Vogt and Paxinos, 2014).

The mPFC has a central role in regulating cognitive processes, both in humans and other mammals. Recent studies using perturbation of the mPFC activity in mice have shown that mPFC is involved in sensory processing, motor planning, emotional regulation, reward, attention, working memory, decision making, long and short-term memory, and social behaviors (Le Merre et al., 2021). Such diverse sets of functions are sustained by a dense pattern of reciprocal connectivity with other cortices, thalamus, subcortical and brainstem regions. Indeed, a recent study in mice found that it is possible to differentiate the mPFC from other cortices based on corticocortical and thalamic connectivity patterns. The mPFC has the highest proportion of feedback projections (Harris et al., 2019).

The HPC and mPFC are connected by both polysynaptic (indirect) and monosynaptic (direct) projections (Jin and Maren, 2015). This pathway seems to be conserved throughout different mammalian groups, as shown by studies in rats (Jay et al., 1989, 1996; Hoover and Vertes, 2007), mice (Tripathi et al., 2016), cats (Irle and Markowitsch, 1982; Cavada et al., 1983), and monkeys (Rosene and Van Hoesen, 1977). The HPC afferents originate in the intermediate and ventral CA1 and in the proximal limb of the subiculum. It then courses through the alveus, following dorsal and rostral by the ipsilateral fimbria and fornix. The fibers continue rostroventrally through the medial part of the lateral septum and nucleus accumbens, reaching the infralimbic (IL), prelimbic (PL) the anterior cingulate cortices (Jay et al., 1989; Jay and Witter, 1991). The IL, PL and ACC form the rodent medial prefrontal cortex.

**FIGURE 1:**
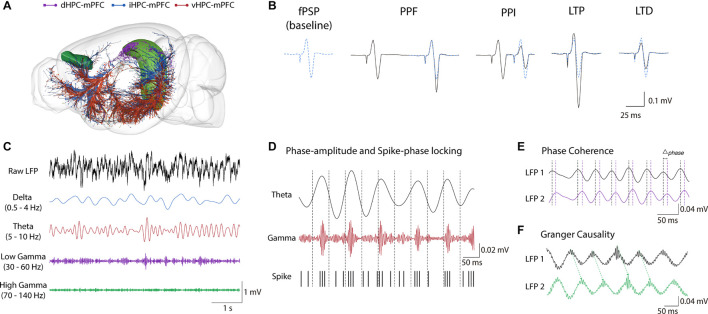
Hippocampus-mPFC anatomical and electrophysiological measures. **(A)** Distinct HPC projections along the septo-temporal axis. Purple: dHPC; Blue: iHPC; Red: vHPC (brain sites adapted from the Brain Explorer, Allen Institute). **(B)** Electrophysiological measures of synaptic plasticity in HPC-mPFC pathway. From Left to right: typical fPSP baseline of the iHPC-mPFC pathway, short-term plasticity (PPF and PPI), and long-term plasticity (LTP and LTD) representatives. Paired-pulse stimulations facilitate or inhibit fPSPs response in longer and shorter interval stimulus, respectively. High-frequency stimulation induces LTP while low frequency typically induces LTD. **(C)** Representative traces of LFPs. Top to down: Raw LFP followed by band-filtered signal in the oscillatory activity commonly investigated in HPC-mPFC (Delta: 0.5–4 Hz, Theta: 5–10 Hz, Low-Gamma: 30-60 Hz, High-Gamma: 70-140 Hz). **(D)** Phase-amplitude and Spike-phase locking. In the example: Theta coupling to Gamma amplitude and neuronal spikes. These methods measure how the amplitude of a fast activity or the firing rate in one region is modulated by phase of the oscillatory activity in another region **(E)** Synchrony measures. In the example: Phase difference (Δ_phase_) between theta oscillatory activity across time. Synchrony methods such as spectral and phase coherence measure the consistency of Δ_phase_ between two signals over time. **(F)** Granger Causality. Granger Causality is inferred when the future values of a signal are predicted using prior values of another signal. fPSP, field post-synaptic potential; PPF, paired-pulse facilitation; PPI, paired-pulse inhibition; LTP, long-term potentiation; LTD, long-term depression; LFP, local field potential.

The hippocampal innervation to the PFC presents differential patterns between the ventral and dorsal PL region. While the ventral portion receives dense projections in the layers II-VI, the dorsal portion receives less dense inputs, mainly present in the layers V-VI (Jay and Witter, 1991; Thierry et al., 2000). Interestingly, ventral HPC inputs make a similar connection onto cortico-cortical and cortico-amygdalar pyramidal neurons in superficial layers of the IL cortex, but make a preferential connection onto cortico-cortical over cortico-pontine neurons in deep layers of IL and PL (Spellman et al., 2021). It has also been demonstrated that HPC targets different types of interneurons in the PFC, presenting differential axon collateral projections. Part of the HPC neurons that project to somatostatin positive interneurons also project to the contralateral CA1, while HPC principal cells that target parvalbumin neurons tend to send projections also to the nucleus accumbens (NAc) (Sun et al., 2019). Furthermore, it is also proposed that differences in the microcircuitry of the target interneurons could explain differential electrophysiological properties of the intermediate (iHPC) and ventral (vHPC) routes (see section 4). It is also clear from the anatomical studies a differentiation between the projections of the dorsal, intermediate, and ventral thirds of the HPC. While there is no projection from the dorsal HPC, the intermediate third of the HPC projects moderately to the infralimbic area, light projection to the prelimbic, and scarce projections to the anterior cingulate area. The ventral part of CA1 projects moderately to the dorsal infralimbic, dense projections to the prelimbic area, and moderate projections to the anterior cingulate area (Jay and Witter, 1991; Cenquizca and Swanson, 2007). Based on this differential projection, electrophysiological patterns, and the aforementioned molecular profile of the hippocampal septo-temporal axis, we adopted the nomenclature dividing the HPC into the dorsal hippocampus (dHPC), iHPC, and vHPC regions (Figure 1A).

## Hippocampus-Prefrontal Cortex Synaptic Efficacy

Synaptic efficacy can be measured by recording postsynaptic potentials (PSP) or postsynaptic currents (PSC) from neurons in culture or in the brain tissue. In *in vivo* and freely behaving preparations, field PSPs (fPSP) are the primary electrophysiological measurement used to investigate synaptic efficacy of a given pathway (Manahan-Vaughan, 2018a, b). The fPSP is evoked when a stimulus in the presynaptic population of neurons generates a depolarization of postsynaptic targets, which can be detected if sufficient extracellular current flows to the recording electrode referenced to an isoelectric ground (Manahan-Vaughan, 2018a). Although field postsynaptic amplitude responses usually relate to the synchronization and spiking activity of target neurons, fPSPs result from the contribution of all synaptic currents flowing through the extracellular space (Manahan-Vaughan, 2018a).

Field PSPs in the mPFC induced by HPC stimulation were first described by Laroche et al. (1990). The authors originally described that CA1 stimulation elicited a characteristic biphasic potential recorded extracellularly in the prelimbic area of mPFC and an excitatory response in single-unit spike recordings (50/120 units). The HPC-mPFC fPSP consists of an initial positive wave followed by a large negative wave between 15 and 22 ms interval latency (Laroche et al., 1990; Takita et al., 1999; Izaki et al., 2003b; Takita et al., 2010; Lopes-Aguiar et al., 2013; Bueno-Junior et al., 2017; Esteves et al., 2017). The negative wave is associated with the synchronous discharge of mPFC neurons since excitatory single–unit responses coincide with the negative wave component of fPSP (Laroche et al., 1990). Indeed, the long latency response is compatible with the estimated slow conduction velocity of fibers (0.6 ms^–1^) and the latency of antidromic stimulation of HPC-mPFC (Ferino et al., 1987).

## Hippocampus-Prefrontal Cortex Synaptic Plasticity

Synaptic plasticity is the ability of nerve cells to modify the efficacy of synaptic transmission, which can be induced either by direct electrical stimulation or by environmental experience (Ho et al., 2011). In general, synaptic plasticity can be divided into two categories time-related: short-term plasticity and long-term plasticity, which can last from milliseconds to minutes or hours to days, respectively (Citri and Malenka, 2008). While short-term synaptic plasticity is related to transient behavioral changes, such as short-term memory and adaptations in sensory pathways, long-term synaptic plasticity is associated with long-lasting behaviors, such as long-term memory, sleep-wake cycle, or even maladaptive behaviors (Romcy-Pereira and Pavlides, 2004; Romcy-Pereira et al., 2009; Godsil et al., 2013).

Experimentally, short-term synaptic plasticity in the HPC-mPFC pathway can be assessed by two pulse stimuli in the presynaptic terminal, which can induce paired-pulse facilitation (PPF) or paired-pulse inhibition (PPI). PPF occurs when two pulse stimuli in the presynaptic terminal enhance the second fPSP amplitude, while PPI represents the reduction of second fPSP (Zucker and Regehr, 2002). Long-term potentiation (LTP) and long-term depression (LTD) are the most extensively studied forms of long-term plasticity in the HPC-mPFC pathway and reflect an increased or decreased neural response, respectively (Laroche et al., 2000). Commonly, the protocol required to induce LTP in the HPC-mPFC projection consists of high-frequency stimulation, while the LTD protocol consists of low-frequency stimulation (Manahan-Vaughan, 2018b). HPC-mPFC LTP and PPF *in vivo* were first described by Laroche et al. (1990), who observed a significant and persistent potentiation of fPSPs for hours (Laroche et al., 1990; Jay et al., 1996; Esteves et al., 2017). More recently, Abraham’s group showed the persistence of HPC-mPFC LTP up to 20 days (Taylor et al., 2016; Figure 1B).

Interestingly, short-term and long-term plasticities in the HPC-mPFC pathway depend on the origin of the HPC projections (Takita et al., 2013). Projections from the vHPC show PPF and PPI peak latencies shorter when compared to projections from iHPC (Izaki et al., 2001, 2002). Moreover, iHPC-mPFC PPF responses are stronger than vHPC-mPFC PPF responses for similar paired-pulse stimulation intervals (Kawashima et al., 2006). The exact mechanism of different functional activities between both pathways is not fully understood. However, Takita et al. (2013) proposed that heterosynaptic circuits in mPFC could provide an explanation to these differences in short-term plasticity (Takita et al., 2007, 2013). Regarding long-term plasticity, iHPC-mPFC is more malleable to long-term plasticity protocols than vHPC-mPFC (Takita et al., 2013). For example, in the iHPC-mPFC pathway, LTP induction is more susceptible to stimulus intensity variation, and previously induced LTP prevents LTD induction, and vice versa (Izaki et al., 2003a; Takita et al., 2010, 2013). Similarly, LTP induction in vHPC-mPFC increases a previously weak LTP and PPF response in iHPC-mPFC. Furthermore, electrolytic lesions in vHPC-mPFC attenuate iHPC-mPFC LTP (Kawashima et al., 2006). These results indicate that iHPC-mPFC and vHPC-mPFC provide distinct but convergent inputs to the mPFC long-term plasticity (Kawashima et al., 2006). Remarkably, HPC-mPFC pathways are related to different behavioral aspects, especially in working memory (Laroche et al., 2000). While bilateral lesions of iHPC interfered with working memory in a delayed alternation task on the order of a few seconds, bilateral lesions of vHPC did not (Izaki et al., 2008). However, vHPC-mPFC is important for longer aspects of working memory (Floresco et al., 1997; Wang and Cai, 2006). In fact, gamma power elevation in mPFC required for working memory is related to LTD induction in vHPC-PFC (Izaki et al., 2004).

## Hippocampus-Prefrontal Cortex Functional Connectivity

Functional connectivity between the HPC and mPFC can be inferred from electrophysiological recordings of spike activity and local field potentials (LFP) during spontaneous behaviors. There are several methods to measure inter-area interaction (Figures 1C-F). Generally, these methods use electrophysiological time-series of different regions to quantify the statistical dependencies of neuronal activity over time (Buzsáki et al., 2012; Sigurdsson and Duvarci, 2016).

It is hypothesized that slow oscillations synchrony, such as delta and theta, provides a mechanism to coordinate network activity. These oscillations are transmitted with minimum phase delays between distant brain regions, allowing the coordination of neuronal spikes and local fast oscillatory activity (Roy et al., 2017). During active and exploratory behavior, HPC activity is dominated by theta oscillation (4-12 Hz) (Buzsáki, 2002), which is generated in the HPC-medial septum network but is a global rhythm recorded in various brain regions, including the mPFC. To date, however, there is no clear evidence whether the mPFC theta is a local oscillation of prefrontal neurons entrained by the HPC or is a measure of HPC volume conduction. Phase-locking of prefrontal spikes and gamma activity to theta oscillation and reduction of this phase-locking under inhibition of HPC projections indicate that theta oscillation can be driven by the hippocampus (O’Neill et al., 2013). In addition, theta coherence is attenuated monotonically as a function of distance from the hippocampus and there is no precise current source density estimate detected in the theta band in the parietal area overlying the hippocampus, suggesting that theta can be volume conducted to cortical areas (Sirota et al., 2008). Despite the controversy, theta oscillation is essential for HPC-mPFC communication. Theta synchrony in the HPC-PFC is dynamically modulated during spatial working memory tasks, and phase locking of PFC units to hippocampal theta and hippocampal gamma oscillations is increased during correct choices in a spatial working memory task (Benchenane et al., 2010). Although the majority of studies investigated theta synchrony between the dHPC and the mPFC, theta synchrony is more robust between the mPFC and vHPC, which is consistent with the anatomical pathways (Adhikari et al., 2010; O’Neill et al., 2013) and has been related to fear and anxiety behaviors.

Synchrony between LFP rhythms in the HPC-PFC pathway has also been described in the gamma frequency (30-80 Hz) and it is thought to support the formation of neuronal assemblies coordinating excitatory spike activity into gamma cycles (Jung and Carlén, 2021). It has been postulated that inter-area functional connectivity and transfer of information can be coordinated by gamma oscillations (Fries, 2015). Gamma coherence in the dHPC-PFC, for example, increases with the learning of spatial reference memory (Yamamoto et al., 2014). Inter-areal brain connectivity can also be measured by cross-frequency coupling (CFC), which quantifies the interaction between oscillatory activities in different frequency bands. Phase-amplitude coupling (PAC) is a measure of CFC that estimates the statistical dependencies of the phase of a slower oscillation and the amplitude of a faster rhythm (Nandi et al., 2019). This oscillatory coupling is proposed to be a mechanism of brain coordination across regions. In the HPC-PFC pathway, it has been described that the HPC theta modulates the envelope of PFC gamma (Sirota et al., 2008; Tamura et al., 2017) and the HPC gamma ( > 60Hz, reflecting population spike activity) induces postsynaptic membrane fluctuations in the theta frequency (Nandi et al., 2019). Theta-gamma PAC in the PFC is also related to improved cognitive performance and spatial working memory (Tamura et al., 2016, 2017).

Theta and gamma activity dominates the HPC-PFC interactions during active behavior. However, distinct oscillatory patterns coordinate communication between the HPC and neocortex during passive behaviors, especially during sleep (Jung and Carlén, 2021). According to the two-stage model of memory consolidation (Buzsáki, 1989), recently encoded representations are gradually transferred from the hippocampus to cortical regions, such as the PFC, during offline behavioral states (i.e. resting or sleeping) (Buzsáki, 2015). Recent studies demonstrated that fine temporal coordination between distinct hippocampal-cortical oscillatory patterns occurs during non-rapid-eye movement (NREM) sleep. Particularly, the coordination between hippocampal sharp-wave ripples (SWR), a well-described high-frequency oscillation registered in CA1 (Buzsáki, 2015), thalamic-cortical spindles, and slow cortical oscillations seems to be critical for this process (Maingret et al., 2016; Latchoumane et al., 2017). Accordingly, depolarization of mPFC neurons was observed synchronously to hippocampal place-cells reactivation during SWR events (Nishimura et al., 2021). Although there is plenty of evidence supporting that ripples trigger the propagation of memory traces from the hippocampus towards the neocortex, the idea of a unidirectional modulation was challenged by recent findings in animal models (Abadchi et al., 2020) and humans (Helfrich et al., 2019). Furthermore, the HPC-PFC dialog during SWRs is accompanied by the inhibition of many diencephalic, midbrain, and brainstem regions. This suggests a possible prioritization of the HPC-PFC interaction by silencing subcortical inputs (Logothetis et al., 2012). There is evidence suggesting that SWRs are endogenous candidates for promoting both LTP (Sadowski et al., 2016) and synaptic depression (Norimoto et al., 2018), but it remains unclear how they regulate the HPC-PFC communication. Particularly, during awake, SWR occurs in periods of post-consummatory behavior with reward-associated dopaminergic activity. The stimulation of hippocampal dopaminergic fibers from the midbrain increases reactivation during SWRs (McNamara et al., 2014). Dopamine also induces the facilitation of SWRs and is thought to reorganize cell assemblies during these events (Miyawaki et al., 2014). During sleep, SWRs are usually detected when cholinergic and noradrenergic levels are reduced. Indeed, the activation of septal-hippocampal cholinergic neurons suppresses SWR (Vandecasteele et al., 2014) and norepinephrine modulates its induction (Ul Haq et al., 2012). Although these neurotransmitters modulate SWR, there is no clear understanding of how they affect the HPC-PFC coordination.

In summary, the interactions between HPC and mPFC are dynamically modulated in different temporal scales, according to environmental and cognitive demands. Neuromodulatory systems play an essential role in regulating both synaptic plasticity and network coupling, enabling an adequate dynamical communication between hippocampal and cortical circuits. In the following sections, we will review studies demonstrating the effects of the main neurotransmitter systems in long- and short-term HPC-PFC synaptic plasticity and its functional connectivity.

## Neuromodulation

### Acetylcholine

**FIGURE 2:**
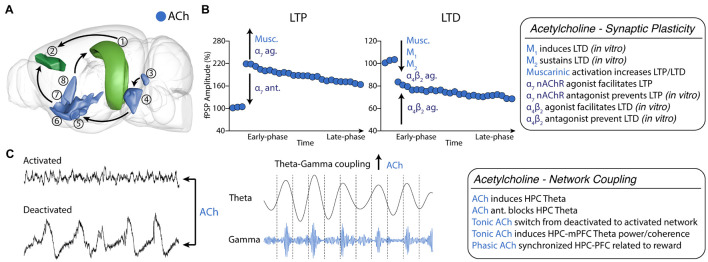
Cholinergic neuromodulation of HPC-mPFC communication. **(A)** HPC-mPFC pathway and cholinergic circuits. (1) Hippocampus CA1/Subiculum; (2) Prelimbic prefrontal cortex; (3) Laterodorsal tegmental nucleus; (4) Pontine reticular nucleus; (5) Magnocellular nucleus; (6) *Substantia innominata*; (7) Diagonal band nucleus; (8) Medial septal nucleus. Blue: main cholinergic anatomical structures (brain sites adapted from the Brain Explorer, Allen Institute). **(B)** Muscarinic and nicotinic receptor activation bidirectionally affects long-term synaptic plasticity, increasing LTP and LTD induction. **(C)** Tonic cholinergic modulation is essential to switch from a deactivated state to an activated state. In addition, modulation of muscarinic activation promotes theta-gamma PAC in HPC-mPFC. Ach, Acetylcholine; LTP, long-term potentiation; LTD, long-term depression; ag, agonist; ant., antagonist.

Acetylcholine (ACh) is one of the main neuromodulators of the central nervous system playing an essential role in attention, regulation of the sleep-wake cycle, learning, and memory (Dannenberg et al., 2017). The brain has two major cholinergic projections: the basal forebrain and the brainstem cholinergic system. The basal forebrain cholinergic system includes the nucleus basalis of Meynert (nucleus basalis magnocellularis in rodents), substantia innominata (NB/SI), the medial septal nucleus, and the horizontal and vertical limbs of the diagonal band of Broca. These regions modulate learning, memory, synaptic plasticity, arousal, and attention (McCormick, 1993; Leanza et al., 1996; Villano et al., 2017). The brainstem cholinergic system comprises the peduncolopontine nucleus and the laterodorsal pontine tegmental nucleus, which has been described as part of the ascending reticular activating system. This system is implicated in the regulation of rapid eye movement (REM) sleep, wakefulness, and vigilance (Shouse and Siegel, 1992; Datta and Siwek, 1997). In rodents, HPC receives cholinergic inputs from the septum-diagonal band complex, whose fibers project to hippocampal subfields and most cell types, including pyramidal cells, granule cells, interneurons, and hilar neurons (Frotscher and Léránth, 1985; Woolf, 1991). The ventral regions of the mPFC (prelimbic and infralimbic), on the other hand, receive strong projections from the horizontal and diagonal band of Broca and only a few projections from the nucleus basalis (Chaves-Coira et al., 2018; Figure 2A).

ACh acts primarily by activating two types of membrane receptors, the G protein-coupled muscarinic acetylcholine receptors (mAChRs) and the ligand-gated nicotinic acetylcholine receptors (nAChRs). Muscarinic acetylcholine receptors (mAChRs) are divided into five subtypes (M_1_-M_5_). Of these receptors, M_1_, M_3_, and M_5_ are excitatory and are coupled with the G_q/11_ family of G proteins, while M_2_ and M_4_ are inhibitory and are coupled with the G_i/o_ family (Volpicelli and Levey, 2004). The M_1_ and M_2_ subtypes are the most abundant mAChRs in the brain (Volpicelli and Levey, 2004). While the M_1_ receptor is localized post-synaptically and is expressed abundantly in the cerebral cortex and HPC, the M_2_ mAChR is an inhibitory autoreceptor localized mainly on large cholinergic interneurons (Levey et al., 1991; Crook et al., 2001). In contrast, nAChRs belong to the family of the ligand-gated ion channels. There are 16 nAChRs subunits identified (α_1_-α_10_, β_1_-β_4_, γ, δ, ε), which can combine in multiple forms of receptors according to the brain region, neuronal subtype and animal developmental stage. The homomeric α_7_ nAChRs and the heteromeric α_4_β_2_ are the most abundant in the adult mammalian brain and are highly expressed in the HPC and mPFC (Gotti et al., 2007).

#### Synaptic Plasticity

Our understanding of the cholinergic modulation of synaptic plasticity in the HPC-mPFC pathway comes mostly from animal studies performed *in vitro* and *in vivo*. The work of Parent et al. (2010) describing a new preparation that allowed the identification and stimulation of pathway-specific ventral hippocampal inputs to neurons of the prelimbic cortex was essential for studying the HPC-mPFC pathway *in vitro*. Using this preparation, Wang and Yuan described that bath application of carbachol (an unspecific AChRs agonist) resulted in a pronounced decay of evoked fPSP (acute phase) that returned to baseline after ∼40 min in cortico-cortical stimulation. However, in vHPC, fPSP was maintained in 75% of the baseline response, characterizing an LTD induction (Wang and Yuan, 2009). They also verified that M_1_ and M_2_ antagonists contributed to the acute phase of synaptic depression, while M_2_ was more related to long-term suppression. Caruana et al. (2011) replicated those findings and showed that a specific prolonged (450 pulses) low-frequency stimulation (1 Hz) induced an LTD form that is dependent on the M_1_ receptor and does not depend on NDMAr.

In a series of elegant experiments, Maksymetz et al. (2019) investigated specific regional inputs to the prefrontal cortex using *in vitro* electrophysiology and optogenetics. First, they reproduced the previous carbachol data using a more specific muscarinic agonist to induce LTD in layer II/III to layer V fPSPs in the prelimbic cortex (Ghoshal et al., 2016). Following, they used an optogenetic approach to stimulate specific afferent projections. Interestingly, the M_1_-induced LTD was specific to the basolateral amygdala or the vHPC projections and produced only mild effects in the mediodorsal nucleus of thalamus projections to the prelimbic cortex. Also, using a viral-mediated selective deletion of M_1_ receptors from glutamatergic pyramidal neurons in the mPFC, they demonstrated that the muscarinic induced LTD in the vHPC-mPFC is dependent on the postsynaptic expression of M_1_ receptors.

Our group pioneered the i*n vivo* investigation of the cholinergic modulation of the iHPC-mPFC synaptic plasticity. We first demonstrated that intraperitoneal administration of the M_1_ preferential agonist pilocarpine (Pilo) potentiated the late-phase of iHPC-mPFC LTP (Lopes Aguiar et al., 2008). Following, we investigated the effects of Pilo on iHPC-mPFC LTD. We demonstrated that previous administration of pilocarpine converted a transient cortical depression into a robust and stable LTD. Importantly, we demonstrated that iHPC-mPFC LTD induction is dependent on NMDA receptors since the selective antagonist AP7 blocked the pilocarpine-induced LTD conversion and the induction of a strong LTD using a supra-threshold protocol (Lopes-Aguiar et al., 2013). The effects of muscarinic activation on iHPC-mPFC synaptic plasticity were confirmed in a later study using intracerebroventricular (i.c.v.) administration of Pilo. However, i.c.v. Pilo administration mainly potentiated LTP induction (Ruggiero et al., 2018). In the same work, we showed that lithium - a drug that interacts with downstream targets of M_1_ signaling - dampened the muscarinic effects of Pilo on LTP, but enhanced on LTD (Ruggiero et al., 2018). Taken together, these results show a bidirectional modulation of iHPC-mPFC synaptic plasticity by M_1_ activation. Importantly, in neither of these studies, cholinergic activation altered basal synaptic transmission, indicating that *in vivo* effects are mainly mediated by downstream targets related to synaptic plasticity.

nAChRs also modulate synaptic plasticity in brain areas intrinsically related to reward and addiction, such as the ventral tegmental area (Mansvelder and McGehee, 2000; McKay et al., 2007). Particularly, in the vHPC-mPFC pathway of intact animals under urethane anesthesia α_7_ receptor agonism facilitates LTP at lower doses and blocks LTP at higher doses (Stoiljkovic et al., 2016). A recent *in vitro* study further elucidated the role of nAChRs on the vHPC-mPFC pathway, using a spike-timing-dependent plasticity (STDP) protocol that induces only a transient increase in excitatory postsynaptic current (Sabec et al., 2018). The authors observed LTP induction or blockade in the presence of α_7_ nAChRs agonist or α_7_ nAChRs antagonist, respectively. On the other hand, STDP induced LTD in the presence of a α_4_β_2_ receptor agonist and was prevented by co-application of a α_4_β_2_ antagonist. In addition, α_4_β_2_ nAChRs LTD was blocked by gabazine, which suggests that GABAergic neurons might intermediate the cholinergic action. Interestingly, neither α_7_ nor α_4_β_2_ nAChRs agonists affected synaptic transmission by themselves (Figure 2B).

#### Functional Connectivity

The cholinergic modulation of theta rhythm is well known for decades. Numerous studies have demonstrated that acetylcholine, cholinesterase inhibitors, and cholinergic agonists can induce hippocampal theta oscillation while antagonists block it (reviewed by Nuñez and Buño, 2021). Cholinergic induction of theta is largely mediated by the activation of M_1_ mAChRs in hippocampal pyramidal neurons (Ovsepian et al., 2004), although nAChRs may also play a role through the basal forebrain cholinergic projections to hippocampal interneurons (Griguoli et al., 2010). In the neocortex, cholinergic inputs exert an activation effect, bringing membrane polarization closer to firing threshold, probabilistically increasing neurotransmission and neuronal responses that are critical for higher arousal states such as awake and REM sleep (Steriade, 2004). This activation is done directly through the NB/SI projections or indirectly through the brainstem cholinergic system afferents to the thalamus and the NB (Steriade, 2004). Activation of both the brainstem or forebrain cholinergic systems disrupts the main oscillatory rhythms of the non-REM sleep (delta oscillation, slow oscillation and spindles) and induces the occurrence of fast oscillatory activity (20-60 Hz) that are prominent during awake and REM sleep and involves thalamo-cortical and cortical circuits (Steriade, 2004).

Under urethane anesthesia, rodents show two distinct spontaneous oscillatory patterns (states): a deactivated state characterized by delta and slow oscillation activity; and an activated state, described by fast oscillatory activity and hippocampal theta (Clement et al., 2008; Pagliardini et al., 2013a, b). Clement et al. (2008) showed that this brain state alternation depends on cholinergic and muscarinic activation. Inactivation of the forebrain cholinergic system disrupted spontaneous alternation (i.e., prolonged the deactivated states), while stimulation of the brainstem cholinergic system immediately produced activated patterns (Clement et al., 2008). In addition, we have recently shown that distinct coupling occurs between the HPC-mPFC during each state. In the deactivated state, we observed a coherence peak in the 0.5-2 Hz band, with the mPFC leading the HPC, while in the activated state, there was a peak in theta with the HPC leading the mPFC and a time lag consistent to data of freely-moving animals (Lopes-Aguiar et al., 2020). We also showed that mAChR activation by pilocarpine dose-dependently increased activated over deactivated states (Lopes-Aguiar et al., 2013). Activated states could also be induced by i.c.v. administration of nicotine. In this case, the increase observed in gamma and beta rhythms were lower compared to muscarinic activation (Bueno-Junior et al., 2012). Similar effects, showing decrease in delta and increase in theta and gamma bands were observed in various cortical areas in freely moving animals after cholinergic modulation (Cape et al., 2000). Interestingly, we further showed an increase in iHPC-mPFC gamma coherence following muscarinic activation (Lopes-Aguiar et al., 2013; Ruggiero et al., 2018). Muscarinic modulation was also shown to promote PAC between HPC theta and mPFC low-gamma activity (Ruggiero et al., 2018). Theta-gamma coupling is also produced in the PFC following phasic acetylcholine release induced by the detection of environmental cues (Figure 2C). Finally, M1 blockade disrupted theta-low gamma coupling in the mPFC (Howe et al., 2017).

### Dopamine

Dopamine (DA) is the most extensively studied modulator of HPC-mPFC synaptic plasticity. Dopaminergic transmission is involved in numerous cognitive, emotional, and motor functions (Tritsch and Sabatini, 2012; Speranza et al., 2021), and impairments of this system are implicated in neuropsychiatric disorders, such as schizophrenia and major depression (Goto et al., 2010; Grace, 2016).

Dopamine is produced in midbrain and hypothalamic neurons. In the midbrain, the ventral tegmental area (VTA) is the primary source of afferents to limbic and cortical structures. There are five subtypes of dopamine receptors (D_1_-_5_) identified to date. They are grouped into two main classes: D_1_-like and D_2_-like. All DA receptors are G-protein coupled receptors (GPCR) which generally activate (via Gs/olf: D_1_-like) or inhibit (via Gi/o: D_2_-like) adenylate cyclase and protein kinase A (PKA) pathway (Beaulieu and Gainetdinov, 2011). The mPFC receives dense projections from the VTA, while the HPC receives more sparse terminals (Santana and Artigas, 2017; Edelmann and Lessmann, 2018). The expression of D_1_ receptors in the mPFC is more densely concentrated in the deep layers (V-VI), which also receives limbic excitatory afferents. In contrast, the expression of D_2_ receptors is preferentially localized in the superficial cortical layers (I-III), largely comprising intracortical connections (Berger et al., 1991; Gaspar et al., 1995; Santana and Artigas, 2017). In the HPC, there is a gradient of D_1_ and D_2_ expression across the dorsoventral axis, in which the ventral region aspect shows greater expression than the dorsal part (for a review, see Edelmann and Lessmann, 2018). Remarkably, VTA terminals in the mPFC occur in both dendritic shafts and spines and present proximity with HPC excitatory afferents forming synaptic triads (Carr and Sesack, 1996). This structural characteristic intriguingly suggests a relationship between DA, HPC, and mPFC.

**FIGURE 3:**
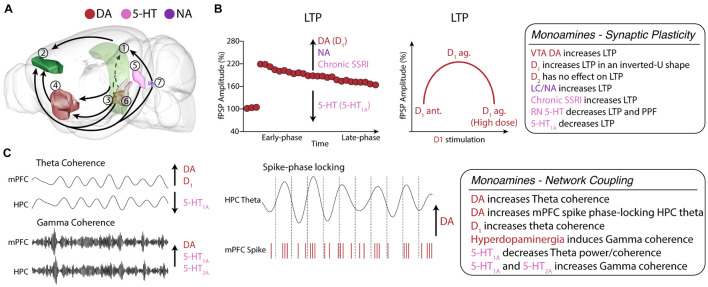
Monoamine neuromodulation of HPC-mPFC communication. **(A)** HPC-mPFC pathway and monoaminergic circuits. (1) Hippocampus CA1; (2) Prelimbic prefrontal cortex; (3) Ventral tegmental area; (4) Nucleus accumbens; (5) Dorsal raphe nuclei; (6) Median raphe nuclei; (7) *Locus coeruleus*. Red: main dopaminergic anatomical structures; Purple: main noradrenergic anatomical structures; Pink: main serotoninergic anatomical structures (brain sites adapted from the Brain Explorer, Allen Institute). **(B)** HPC-mPFC LTP is positively modulated by DA, NA, and chronic SSRI, while 5-HT decreases LTP. In particular, D_1_ activation affects LTP in an inverted U-shaped dose dependence, providing a fine-tune functional regulation in the HPC-mPFC pathway. **(C)** In HPC-mPFC communication, D_1_ and 5-HT_1A_ receptors induce opposite effects in theta coherence, however, both increase gamma coherence. DA, dopamine; 5-HT, serotonin; NA, noradrenaline; SSRI, selective serotonin reuptake inhibitor; LTP, long-term potentiation; LTD, long-term depression.

Although *in vitro* studies demonstrate the dependence of PFC plasticity on DA, they do not reveal such a clear picture of how this modulation occurs (reviewed in Goto et al., 2010) as it has been observed in the intact brain (Jay et al., 2004). For this reason, we will review only studies that preserved the HPC-mPFC and mesolimbic circuits (Figure 3A). Jay et al. (1995) showed that electrical stimulation of the VTA, known to induce cortical DA release, inhibits the neural response of most (73%) mPFC neurons responsive to vHPC stimulation. This finding provided clear electrophysiological evidence for the convergence between vHPC and VTA afferents onto the mPFC, corroborating previous morphological studies. Additionally, they reported that electrical stimulation of the NAc induced antidromic activation of vHPC-responsive mPFC neurons (Jay et al., 1995). This work also suggested a circuit where vHPC controls DA release from the VTA through the PFC and NAc. Other studies confirmed that vHPC stimulation increases DA levels (Blaha, 1997; Floresco et al., 2001). However, the subsequent studies showed that the vHPC could control the VTA even when the PFC is inhibited by tetrodotoxin (Floresco et al., 2001). The control of the HPC over the VTA release is reviewed in Lisman and Grace (2005).

#### Synaptic Plasticity

Gurden et al. (1999) showed that the lesion of DA neurons in the VTA by local injection of 6-hydroxydopamine suppresses LTP in the vHPC-mPFC pathway. The same work showed a strong positive correlation between DA levels in the mPFC and vHPC-mPFC LTP, demonstrating the critical importance of the mesolimbic system and DA in the vHPC-mPFC long-term plasticity. In another study, Gurden et al. (2000) showed that a D_1_ antagonist abolishes vHPC-mPFC LTP, while a D_1_ agonist enhances it. In turn, both D_2_ agonist and antagonist did not affect HPC-mPFC LTP. The dependence of vHPC-mPFC LTP on D_1_-mediated signaling has been replicated several times and is likely the best-known mechanism of modulation of the vHPC-mPFC long-term synaptic plasticity (Jay, 2003; Goto and Grace, 2008; Xu et al., 2016). Importantly, DA seems to modulate both the early and late phases of LTP in the HPC-mPFC synapses, suggesting a role in modulating short-term plasticity. Goto and Grace (2008) showed that theta-burst stimulations (TBS) induced short-term potentiation of the dHPC-mPFC fPSP and that D_1_, but not D_2_, antagonists could block this induction. Together, these findings suggest that D_1_-mediated signaling is critical for short-term and long-term synaptic facilitation of the HPC-mPFC pathway.

Another finding of Gurden et al. (2000) was that D_1_ overstimulation could worsen LTP. Therefore, indicating that D_1_ modulation of HPC-mPFC plasticity follows an inverted U-shape relationship, where low levels of D_1_ activation (e.g., D_1_ antagonism or VTA lesion) suppresses LTP, then an optimal level of DA is associated with increased LTP, but high doses would impair LTP again. Remarkably, the same inverted U-shape association of D_1_ and PFC function has been well established for *in vitro* plasticity and for working memory performance (Goldman-Rakic et al., 2000; Seamans and Yang, 2004; Williams and Castner, 2006; Goto et al., 2007; Kolomiets et al., 2009; Figure 3B).

#### Functional Connectivity

Despite showing greater concentrations onto the PFC and striatum, DA afferents are widespread throughout the brain, suggesting a role in modulating network activities. DA levels are enhanced during working memory tasks (Phillips et al., 2004; Robbins and Arnsten, 2009). Benchenane et al. (2010) showed an increase in iHPC-mPFC theta coherence throughout a spatial working memory task, especially during delayed periods. Next, they showed in the anesthetized rat that the local infusion of DA in the mPFC mimicked the electrophysiological effects observed during memory performance, such as iHPC-mPFC theta coherence and phase-locking of mPFC neurons to iHPC theta field. This finding is remarkable because it suggests that DA facilitation of HPC inputs promotes greater synchrony in the theta band, which comprises an oscillatory activity generated in the hippocampus, but is essential for many PFC-associated behavioral functions (Benchenane et al., 2011). In support, Xu et al. (2016) reported that i.c.v. injection of a D_1_ antagonist does not change vHPC and mPFC theta power but decreases both vHPC-mPFC theta coherence, directionality, and theta-slow gamma coupling (Xu et al., 2016). Dzirasa et al. (2009), using transgenic mice knockout for the DA transporter, which presents hyperdopaminergia, reported increased gamma coherence between dHPC and mPFC. Recently, Gener et al. (2019) showed in freely moving mice that D_2_ agonism reduced dHPC-mPFC theta power and synchrony but increased them in delta. They also showed decreases in beta and gamma power, all paralleled to reduced locomotion. Then, D_2_ antagonism with haloperidol reversed the effects on beta, gamma and theta power and theta synchrony (Figure 3C).

### Noradrenaline

Noradrenaline (NA) is produced in the brainstem in specific locus coeruleus (LC) neurons. It has widespread terminals onto the forebrain, and it is proposed to be one of the most important brain systems to modulate arousal and attention (Sara, 2009). NA has three main types of receptors, α_1_ (Gq GPCR), α_2_ (Gi/o GPCR), and β (Gs GPCR), which are subdivided into three subtypes (Marzo et al., 2009). Importantly, NA receptors also have an affinity with epinephrine, a hormone released in the body during stress, suggesting that NA transmission is associated with stress-related modulation of brain function (Ross and Van Bockstaele, 2021). The PFC expresses NA receptors less densely than DA and in a more distributed manner across layers (Santana and Artigas, 2017). The HPC, in turn, expresses more adrenergic receptors than DA, where it is critical for LTP, especially in the Schaffer collateral pathway (CA3-CA1) (Nguyen and Connor, 2019). NA modulation of synaptic plasticity, including at the HPC and PFC, is reviewed in Marzo et al. (2009) (Figure 3A).

Lim et al. (2010) performed a comprehensive investigation demonstrating a positive link between NA and enhancement of vHPC-mPFC LTP. LC electrical stimulation just before bursts of HFS increased vHPC-mPFC LTP. In contrast, inhibition of the LC by local lidocaine injection decreased LTP. Recently, Takeuchi et al. (2016) showed that DA rather than NA released from the LC might play a more important role in plasticity and memory within the HPC. Nevertheless, Lim et al. (2010) showed that a NA reuptake inhibitor and systemic administration of an α2 antagonist, which disinhibits NA release, also increased LTP. In contrast, α2 agonist and partial lesion of NAergic LC neurons by DSP-4 decreased LTP. Noteworthy, no studies manipulated NA transmission directly in the PFC, and we still do not know the roles of specific adrenergic receptors at the mPFC in the facilitatory effect of NA on HPC-PFC plasticity. Nevertheless, NA exerts positive effects on mPFC-related cognitive functions and HPC plasticity via β-mediated signaling, which generally acts through the PKA pathway (Chamberlain and Robbins, 2013; Nguyen and Connor, 2019), while it modulates HPC and intracortical mPFC LTD mainly via α1 (Marzo et al., 2010). In addition, Lim et al. (2017) showed that NA indirectly modulates vHPC-PFC plasticity via the amygdala, a key region for processing fear and stress. NA-mediated activation and inhibition of the amygdala positively and negatively modulated vHPC-PFC LTP, respectively. Besides the role in stress, Bhardwaj et al. (2014) investigated the effects of neonatal lesion of vHPC, which is described as a model of schizophrenia, and reported a disruption of NA modulation of mPFC corticocortical plasticity via alterations of α1 signaling. Notably, NA and DA interact (Xing et al., 2016; Figure 3B). Therefore another possible mechanism of NA modulation could be indirectly by influencing DA transmission. NA manipulations can increase DA levels, and LC stimulation can activate the VTA (Grenhoff et al., 1993). The interaction between these signaling pathways has been investigated in the HPC and the mPFC (Xing et al., 2016) but still not in the HPC-PFC pathway.

### Serotonin

Serotonin (5-hydroxytryptamine; 5-HT) is produced in specific neurons of the midbrain that comprise the raphe nuclei (RN). The RN has widespread innervations to all the forebrain, and the HPC and PFC are principal targets of these innervations (Azmitia and Jacobs, 1992). The most relevant nuclei regarding HPC and PFC function are the dorsal raphe nucleus (DRN) and the median raphe nucleus (MRN) (Puig and Gener, 2015). The DRN has widespread terminals in the forebrain and is the primary source of serotonin to the PFC, to whom it has reciprocal connections (Puig and Gulledge, 2011; Pollak Dorocic et al., 2014). In turn, the MRN has more specific connections, and it is the primary source of 5-HT to the septohippocampal system (Vertes and Kocsis, 1997). There are seven families of 5-HT receptors (5-HT_1_-_7_) divided into fourteen subtypes. All subtypes are GPCR, except for the 5-HT_3_, which is ionotropic. The most expressed receptors in both structures are the 5-HT_1A_ (Gi/o GPCR) and the 5-HT_2A_ (Gs GPCR). There is a specific profile of expression of the distinct 5-HT receptor throughout neurons. In both PFC and HPC, 5-HT_1A_ is preferentially expressed in the somata and axons, while the 5-HT_2A_ is on the apical dendrites of pyramidal cells (Puig and Gener, 2015). PFC pyramidal neurons show co-expression of both receptors in the same neurons (Amargós-Bosch et al., 2004). Therefore, 5-HT has a significant inhibitory effect on the firing rate but promotes excitatory effects on field potentials (Puig and Gulledge, 2011; Figure 3A).

#### Synaptic Plasticity

The first implication of the 5-HT system in the modulation of HPC-mPFC synaptic function showed that both single and chronic applications fluvoxamine, an antidepressant that acts as a selective serotonin reuptake inhibitor (SSRI), could increase vHPC-mPFC fPSP (Ohashi et al., 2002). Also, the fPSP increases occurred in a dose-dependent manner by acute injection. However, they observed that only the chronic, but not acute, treatment increased vHPC-mPFC LTP (Ohashi et al., 2002). It is well established that SSRI antidepressants only promote their therapeutic effects against depression through chronic treatment, which can begin to manifest up to two weeks after treatment (Kraus et al., 2017). Thus, the distinct modulation of LTP by chronic and acute administration of SSRI may represent a manifestation of long-term mechanisms equivalent to those underlying the clinical effects of antidepressants rather than acute effects of 5-HT receptors activation.

In another study, Ohashi et al. (2003) investigated the effects of the lesion of 5-HTergic neurons by administration of 5,7-dihydroxytryptamine. They showed that 5-HT depletion increased vHPC-mPFC PPF and produced a remarkable increase of LTP, which showed a negative correlation with mPFC 5-HT levels (Ohashi et al., 2003). Little is known about the role of specific 5-HT receptors in the modulation of HPC-mPFC plasticity. Xu et al. (2016) administered an i.c.v. injection of a 5-HT_1A_ agonist and reported increased vHPC-mPFC LTP. This finding is discussed in the scenario where 5-HT_1A_ agonism acts on RN presynaptic terminals inhibiting 5-HT release, which supports the role of 5-HT in suppressing this LTP. However, the drug could also be acting directly on the PFC to some degree, so further studies should address this issue (Figure 3B).

#### Functional Connectivity

5-HTergic neurons project densely to the HPC and PFC and present widespread innervations to the forebrain (Azmitia and Jacobs, 1992). Early studies demonstrated that MRN 5-HT transmission is the main regulator of HPC theta oscillations, the most prominent network activity of this structure (Vertes and Kocsis, 1997; Vertes et al., 2004). Lesion of the MRN and 5-HT inhibition enhances theta, while stimulation decreases it (reviewed in Vertes and Kocsis, 1997). 5-HT has also been shown to decrease HPC gamma *in vitro* (Krause and Jia, 2005; Johnston et al., 2014). 5-HT_1A_ knockout mice exhibit greater HPC and PFC theta power in anxiogenic environments (Gordon, 2005; Adhikari et al., 2010). More recently, the role of 5-HT transmission was investigated in the PFC. Puig et al. (2010) showed that 5-HT modulates fast-spiking interneurons, which are critical for generating brain rhythms. 5-HT infusion in the PFC in anesthetized rats decreased firing rates but increased the frequency of UP states, which are rhythmic periods of excitability. These UP states carry gamma oscillations, so 5-HT increased them consequently. The effects of 5-HT on the HPC and PFC network activities are reviewed in Puig and Gener (2015).

Only recently, the modulation of 5-HT on HPC-PFC network synchrony has been examined. In anesthetized rats, i.c.v. injection of a 5-HT_1A_ agonist increased PFC, but not vHPC, theta power and decreased both slow and fast gamma in the HPC and mPFC. No effects were observed in theta coherence, but they reported an increase in unidirectional gamma coherence from vHPC to mPFC, but not the other way around. 5-HT_1A_ agonism also increased vHPC-mPFC fast gamma coherence but decreased theta-fast gamma coupling. Interestingly, these findings indicate that 5-HT and DA modulate theta coupling to distinct gamma frequencies (Xu et al., 2016). In alert rats, particularly during awake immobility states, 5-HT_1A_ activation decreased power in a broad range of oscillatory bands including theta, beta, slow gamma, and fast gamma activities in both dHPC and PFC, as well as induced a profound decrease in dHPC-mPFC theta coherence. Noteworthy, this study also showed that subsequent administration of 5-HT_1A_ antagonist reversed all of these effects. In turn, 5-HT_2A_ activation produced an enhancement of PFC high-gamma power and HPC-PFC high-gamma coherence. 5-HT_2A_ antagonist profoundly decreased HPC-PFC power of theta, slow and high gamma, and decreased theta coherence, while it dramatically increased HPC-PFC delta power (Gener et al., 2019). Interestingly, the effects of PFC delta power may reflect antipsychotic properties since both 5-HT2A and D2 antagonists increase it while 5-HT2A agonists with hallucinogenic effects decrease it. Also, Kjaerby et al. (2016) showed that presynaptic activation of 5-HT_1B_ in the mPFC suppressed vHPC inputs *in vitro* and reduced both mPFC theta power and anxiety measures in the elevated plus maze (Figure 3B).

### Endocannabinoids

The endocannabinoid (eCB) system is a complex, widespread neuromodulatory pathway in the mammalian brain regulating multiple neural functions involved in cognitive, sensory, and emotional processes (Piomelli, 2003). The eCB system includes cannabinoid receptors, eCB neurotransmitters, and enzymes related to the synthesis and degradation of eCBs (Lu and MacKie, 2016).

**FIGURE 4:**
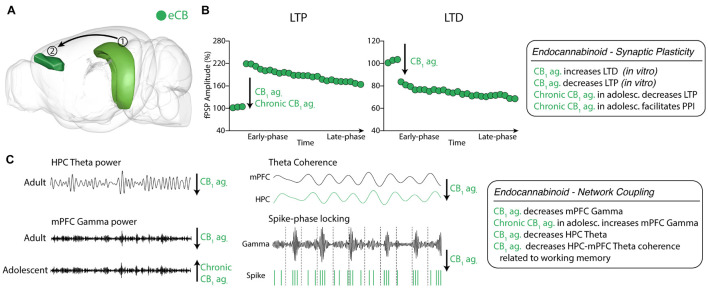
Endocannabinoid neuromodulation of HPC-mPFC communication **(A)** eCB system in HPC and mPFC areas. (1) Hippocampus CA1; (2) Prelimbic prefrontal cortex. Green: eCB neurotransmission in HPC and mPFC (brain sites adapted from the Brain Explorer, Allen Institute). **(B)** CB_1_ activation decreased LTP maintenance in HPC-mPFC pathway. **(C)** CB_1_ agonism reduces HPC theta power and decreases mPFC Gamma power in adults, while chronic CB_1_ agonist treatment during adolescence increased mPFC Gamma power in adults. Also, CB_1_ agonism decreases HPC-mPFC theta coherence during a spatial working memory task and spike-gamma band phase locking in mPFC. eCB, endocannabinoid; LTP, CB_1,_ cannabinoid receptor 1; long-term potentiation; LTD, long-term depression. ag, agonist; ant., antagonist.

The main eCB receptors are the CB_1_ and CB_2_ receptors, primarily expressed in the central nervous system and the peripheral immune cells, respectively (Chen et al., 2017; Kendall and Yudowski, 2017). The CB_1_ and CB_2_ receptors are Gi/Go-coupled receptors which inhibits adenylyl cyclases and voltage-dependent calcium channels, while activating MAP kinases and potassium channels, reducing neurotransmitter release (Howlett et al., 2002). The most abundant eCB neurotransmitters are 2-arachidonoylglycerol (2-AG) and anandamide (AEA), which are post-synaptically, synthesized “on-demand” by 2-arachidonoyl-containing phospholipids and N-arachidonoyl phosphatidyl ethanol, respectively (Pacher et al., 2006). In turn, monoacylglycerol lipase degrades 2-AG, and fatty acid amide hydrolase (FAAH) degrades AEA (Di Marzo et al., 1994; Dinh et al., 2002). In the central nervous system, CB_1_ receptors are fundamentally pre-synaptically expressed both in excitatory and inhibitory synapses in mesocorticolimbic circuits, mediating complex retrograde signaling (Ohno-Shosaku and Kano, 2014). Not surprisingly, the eCB is a fundamental regulator in synaptic plasticity and functional connectivity in the HPC-mPFC pathway (Heifets and Castillo, 2009; Katona and Freund, 2012; Figure 4A).

#### Synaptic Plasticity

*In vitro* CB_1_ activation of mPFC pyramidal neurons decreases baseline excitatory postsynaptic currents, increasing the proportion of cells exhibiting LTD and decreasing the proportion of cells exhibiting LTP. In contrast, CB_1_ antagonism induces the opposite effect (Auclair et al., 2000; Riedel and Davies, 2005). However, little is known about the HPC-mPFC eCB modulation *in vivo* and in freely moving animals (Augustin and Lovinger, 2018). CB_1_ agonists, when administered during adolescence, affect short-term and long-term plasticity in the vHPC-mPFC pathway in adults (Raver et al., 2013; Cass et al., 2014; Renard et al., 2016, 2017a, b). Chronic exposure to CB1 agonist in early adolescent rats facilitates fPSP response in PPI-like protocols susceptible to GABAergic modulation (Thomases et al., 2013; Cass et al., 2014). Indeed, CB_1_ agonist exposure during adolescence reduces mPFC GABAergic transmission *in vivo*, and intra-mPFC GABA-Aα1 receptor positive allosteric modulator restores mPFC transmission (Cass et al., 2014). During adolescence, CB_1_ agonist treatment also reduced the dendritic arborization in pyramidal neurons and postsynaptic levels of PSD95 in mPFC, which is related to impairments of vHPC-mPFC LTP induction (Renard et al., 2016). These results seem to emerge from a developmental impairment of mPFC GABAergic transmission by overactivation of the CB_1_ receptor (Raver et al., 2013; Caballero et al., 2014; Renard et al., 2016, 2017b, a; Figure 4B).

#### Functional Connectivity

Δ9-tetrahydrocannabinol (THC) is the main psychoactive constituent of *Cannabis sativa* (Connor et al., 2021). THC is a partial agonist of CB_1_ and CB_2_ receptors and is an essential pharmacological tool for understanding drug abuse effects and eCB neuromodulation (Connor et al., 2021). Low acute doses of THC (1 mg/kg) decreased the average firing rate of mPFC cells and increased vHPC-mPFC delta (0.3-4 Hz) coherence, without effects on mPFC and vHPC power (Aguilar et al., 2016). However, high THC doses (5 mg/kg) decreased gamma (30-55 Hz) power in mPFC and overall spectral power in the vHPC (Robbe et al., 2006; Nelong et al., 2019). Interestingly, THC chronic exposure during adolescence increases mPFC gamma oscillations *in vitro* and *in vivo* permanently in adult rats while reduces the expression levels of GAD67 in the mPFC (Raver et al., 2013; Renard et al., 2017b).

Like THC effects, acute intraperitoneal treatment with CP55940 (synthetic full CB_1_ agonist) in awake rats decreased firing rates in vHPC and burst activity in mPFC (Robbe et al., 2006; Kucewicz et al., 2011). Moreover, CP55940 decreased the overall power spectrum of vHPC LFP in anesthetized and freely moving rats, mainly on theta, high gamma (62-90 Hz), and ripple oscillations (100-200 Hz) (Robbe et al., 2006; Hajós et al., 2008). Interestingly, the CP55940 effects on vHPC power cannot be explained by firing rate changes. There was no correlation between LFP power and firing rates in vHPC, regardless of CP55940 dose (Robbe et al., 2006). However, CP55940 selectively decreased the incidence of short interspike intervals, which was positively correlated with LFP power changes, suggesting a role of interneurons in vHPC oscillations. Also, CP55940 reduced temporal synchronicity of previously simultaneous recorded cell pairs in vHPC, demonstrating decreased spike coactivation (Robbe et al., 2006).

The CB_1_ agonist effects on HPC activity are associated with mPFC dysfunction as well. The theta power reduction in iHPC induced by CP55940 treatment reduces mPFC gamma (30-100 Hz) power, which could also be related to CB_1_ effects on interneurons (Kucewicz et al., 2011). Indeed, CP55940 reduced firing rates of mPFC in 10-100 ms interspike intervals associated with decreases in gamma oscillations (Kucewicz et al., 2011). Accordingly, CP55940 treatment significantly impaired spatial working memory performance associated with iHPC theta power and gamma (30-60 Hz) reductions (Robbe et al., 2006; Kucewicz et al., 2011). Additionally, CP55940 disrupted iHPC-mPFC theta coherence during the spatial working memory task and impaired spike-locking of mPFC single-units to the local gamma oscillation (Kucewicz et al., 2011). Taken together, these results suggest that CB_1_ agonism effects on HPC-mPFC communication are associated with abolishing the temporal synchrony essential for working memory. However, the complexity of eCB neuromodulation does not allow generalizations about its CB_1_ agonists.

Differently from THC low acute dose treatment, the enhancement of AEA signal by URB597 (FAAH inhibitor) increased mPFC low gamma power (30-55 Hz) without increasing local firing rate (Aguilar et al., 2016). The AEA enhancement reduced vHPC delta power and vHPC-mPFC delta coherence, while THC in low doses increased vHPC-mPFC delta coherence (Aguilar et al., 2016). The mechanisms of the distinct effects of THC and AEA in the HPC-mPFC pathway have not been fully understood, but it is reasonably associated these effects to distinct effects occurs regional specificity of eCB system. URB597 only acts in regions with FAAH activity and will augment AEA levels on-demand, whereas THC widely activates CB_1_ and CB_2_ receptors (Egertová et al., 2003). Besides, the AEA signal affects other receptors, such as transient receptor potential vanilloid 1 (TRPV1) (Di Marzo et al., 2002; Figure 4C).

## Hippocampus-Prefrontal Cortex Neuromodulation in Cognition and Animal Models of Neuropsychiatric Disorders

Hippocampus-prefrontal cortex communication underlies cognitive and behavioral functions that are critically impacted in neuropsychiatric conditions such as schizophrenia, major depression, anxiety disorders, and Alzheimer’s disease (Godsil et al., 2013; Liu et al., 2016; Padilla-Coreano et al., 2016; Sampath et al., 2017). Therefore, a deeper understanding of the HPC-PFC functional connectivity, its regulatory dynamics, and neuromodulatory mechanisms are relevant for advancing towards a better treatment to these conditions.

### Cognition

Several studies report the role of the HPC-mPFC pathway in cognitive processes, such as spatial working memory and memory consolidation (Laroche et al., 2000; Eichenbaum, 2017). It has been argued that the ventral and intermediate HPC-mPFC projections are more related to working memory tasks, while the dorsal HPC is related to memory consolidation. Optogenetic inhibition of vHPC terminals in the mPFC during the encoding phase of a T-maze working memory task results in memory impairments (Tamura et al., 2017). Further, CNO injection into mPFC of subjects expressing hM4D(Gi) DREADD in HPC inactivated HPC-mPFC communication and disrupted memory consolidation (Ye et al., 2017). Similar approaches have been employed to investigate the mechanisms of plasticity in the HPC-PFC circuit. For example, the firing responses in the mPFC after LTP induction in the iHPC-mPFC projections were attenuated by optogenetically controlling the paraventricular/mediodorsal thalamic area (Bueno-Junior et al., 2018). Functionally, several studies report increases in HPC-mPFC oscillatory synchrony during spatial working memory tasks (Jones and Wilson, 2005; Benchenane et al., 2011; Yu and Frank, 2015) and memory consolidation (Paz et al., 2008; Binder et al., 2019). Despite the few works that directly investigated neuromodulation of HPC-mPFC during these cognitive processes, cholinergic neuromodulation of HPC-mPFC seems to be fundamental.

Microdialysis experiments demonstrated that the cholinergic drive increases in the neocortex and hippocampus during cognitive processes (Pepeu and Giovannini, 2004). This indirect evidence may lead us to conjecture that cholinergic tone can modulate the synchronization dynamics between HPC-mPFC during cognitive tasks. Recent evidence has shown that this interaction is more complex, depending on brain state and cognitive demand. First, the traditional view of the acetylcholine neurotransmission as primarily spatially diffuse and slow-acting, in the scales of minutes, has been challenged (Sarter et al., 2009; Sarter and Lustig, 2020). A recent study using *in vivo* amperometry and LFP recordings has helped elucidate the dHPC-mPFC acetylcholine release dynamics showing that tonic and phasic ACh release occurs in both regions, occur in different timescales, and conduct different functions (Teles-Grilo Ruivo et al., 2017).

Interestingly, both modes of transmission occur in a coordinated manner in the HPC and PFC. Tonic ACh release mode is strongly associated with a transition to a high-arousal state and the switch from deactivated to activated. On the other hand, phasic ACh release occurred only during a performance in a cognitive task and was strongly related to reward occurrence. In addition, Maharjan et al. (2018) used viral vector-mediated expression of muscarinic M_4_ receptor coupled to Gi protein, hM4D(Gi) DREADD, to inactivate excitatory neurons in the dHPC and mPFC in a W-track spatial alternation task that tested both working and spatial memory. Their results demonstrated that contralateral dHPC-PFC inactivation with CNO impaired working but not spatial memory in the W-track test. Taken together, these results demonstrate that while coordinated HPC-PFC tonic ACh release is associated with arousal and deactivated states, creating a purposeful context to dynamical coordination between HPC-PFC during cognitive tasks may involve other neurotransmitters (Benchenane et al., 2010). Synaptic plasticity and functional effects of muscarinic stimulation in iHPC-mPFC are associated with enhancement of dopamine release in mPFC (Lopes Aguiar et al., 2008). In addition, D_1_ receptor modulation in HPC-mPFC is critical to performance spatial working memory (Seamans and Yang, 2004). However, as seen before, D_1_ modulation in HPC-mPFC synaptic plasticity occurs in an inverted U-shaped dose dependence, and DA facilitates theta oscillation HPC inputs to mPFC engagement (Gurden et al., 2000; Benchenane et al., 2010). Thus, dopaminergic stimulation plays a role in fine-tuning the information flow in HPC-mPFC.

### Schizophrenia

The HPC-PFC synaptic plasticity and functional connectivity have also been implicated in several animal models of schizophrenia. Goto and Grace (2006) observed an aberrant increase of vHPC-mPFC LTP in the neurodevelopmental model of prenatal exposure to methylazoxymethanol acetate (MAM). In a genetic mouse model of schizophrenia, Sigurdsson et al. (2010) observed a decrease of HPC-PFC theta synchrony correlated with poor spatial working memory performance. Dickerson et al. (2010) found a similar reduction of synchrony in the maternal immune activation (MIA) neurodevelopmental model. Ultimately, Spellman et al. (2015) showed an augmented HPC theta-mPFC slow gamma coupling in wild-type mice correlated to spatial working memory task difficulty, which was even higher in a genetic model of schizophrenia that presented an impaired performance on the task. Similarly, pharmacological animal models of schizophrenia using NMDA receptor antagonists also indicate HPC-mPFC synaptic disruption. Ketamine, an NMDA receptor antagonist, reduced vHPC-mPFC LTP induction in rats, while co-administration of the atypical antipsychotic clozapine counteracted these effects (Rame et al., 2017). Alvarez et al. (2020) showed that early ablation of NMDA receptors in corticolimbic parvalbumin-expressing interneurons of mice impacts vHPC-mPFC connectivity before and after adolescence. While juvenile mice exhibited elevated cortical excitability in an uncoordinated manner relative to the HPC, adult mice showed weaker evoked potentials in mPFC and increased LTD, suggesting impaired functional connectivity during adulthood. In iHPC-mPFC, ketamine-induced aberrant oscillatory coupling in mPFC and alterations in the HPC-PFC synaptic efficacy (Lopes-Aguiar et al., 2020). The authors demonstrated that prior induction of LTP in the CA1-mPFC attenuates these effects, highlighting the importance of high-frequency stimulation as a non-pharmacological alternative strategy to investigate HPC-PFC modulation and help in the prevention or attenuation of cognitive impairments in schizophrenia.

Remarkably, dopaminergic and eCB neuromodulation in HPC-mPFC are associated with the impairments in animal models of schizophrenia. D_2_ agonism or D_1_ antagonism revert aberrant LTP enhancement in the MAM model (Goto and Grace, 2006). However, there is still no evidence for the D_2_ attenuating effect of LTP in a non-pathological brain state. Although most studies focused on the positive modulation of LTP by D_1_ and showed the independence of this phenomenon on D_2_, recent evidence implicates D_2_ signaling in the occurrence of LTD. Banks et al. (2015) applied a TBS protocol to induce LTD in the HPC-PFC *in vitro*. Then, they showed that applying a D_2_ antagonist, but not D_1_, blocked LTD induction. Strikingly, they showed that LTD induction impaired NMDAr, but not AMPAr, synaptic transmission in this pathway. This finding is intriguing because it provides a mechanism that gathers exaggerated DA, hypofunction of NMDAr, and treatment by D_2_ antagonism, which comprises critical neurobiological elements of schizophrenia (Grace, 2016). There is also a complex relationship between eCB neuromodulation and HPC-mPFC (Aguilar et al., 2016; Ruggiero et al., 2017). URB597, a FAAH inhibitor that leads to AEA accumulation, increases mPFC firing rate only in rats submitted to subchronic phencyclidine (PCP) model, while THC does not affect mPFC firing rate following PCP model but decreases firing rate in the control group (Aguilar et al., 2016). The exact mechanism of these effects remains to be elucidated but it is likely related to disruption in other neurotransmission systems (i.e., dopaminergic and GABAergic) induced by PCP, which differently influence eCB effects (Laviolette and Grace, 2006; Volk and Lewis, 2016).

### Major Depression and Anxiety

Alterations in synaptic plasticity are among the main effects of a deleterious experience of stress (Pittenger and Duman, 2008). It is proposed that antidepressants, such as SSRIs, counteract the effects of stress on plasticity across the brain (Pittenger and Duman, 2008; Duman, 2014). This pattern also occurs in the HPC-mPFC pathway. Rocher et al. (2004) exposed rats to an unbalanced elevated platform, which induced a lasting increase in stress-related glucocorticoids, and they observed a marked impairment of vHPC-mPFC LTP. Then, they observed that acute systemic injection of fluoxetine, an SSRI, and the atypical antidepressant tianeptine restored LTP. Interestingly, both drugs did not affect LTP in non-stressed animals. Also remarkable, tianeptine presented a more significant effect than fluoxetine (Rocher et al., 2004). Another study applying the same stress procedure compared the effects of tianeptine and imipramine, a general monoamine reuptake blocker, and observed no significant effect of imipramine on restoring stress-induced effects on LTP (Qi et al., 2009). Nevertheless, although these findings have significant implications for the link between HPC-mPFC plasticity and depression etiology and treatment, tianeptine does not have a specific mechanism of action, so it hinders interpretation. Additionally, there are reports that fluoxetine increases the release of both DA and NA in the cortex (Bymaster et al., 2002), which could also be the actual modulators of plasticity. Moreover, recent evidence indicates that antidepressants may bind directly to tyrosine kinase receptors (TrkB) (Casarotto et al., 2021), which are usually activated by the brain-derived neurotrophic factor (BDNF), which is a pivotal regulator of neural plasticity (Castrén and Antila, 2017). In conclusion, 5-HT modulates HPC-PFC LTP negatively, but antidepressants, including SSRIs, facilitate plasticity, possibly through serotonin-independent pathways.

Among the best studied behavioral functions of HPC-PFC transmission is anxiety. A series of studies using the elevated plus-maze showed that mPFC synchronizes preferentially with the vHPC rather than dorsal parts, especially in the theta frequencies at the environment’s safer zones (closed arms) (Adhikari et al., 2010). Then, they also showed that the mPFC neurons that encode aversion-related information, such as the location in the closed or open arms, are strongly phase-locked to vHPC theta (Adhikari et al., 2011). Similar results were found in the open field. Optogenetic inhibition of vHPC-mPFC terminals decreased both theta synchrony in the anxious environment and expression of anxiety-related behaviors (Padilla-Coreano et al., 2016). More recently, it was shown that sinusoidal optogenetic stimulation of vHPC-PFC terminals at the theta frequency, which mimic more precisely endogenous LFP, induced behavioral avoidance to the safer zones more robustly than by applying single pulses (Padilla-Coreano et al., 2019). Anxiety-related behaviors are also linked to serotonin neuromodulation in the vHPC-mPFC circuit. Knockout mice for 5-HT_1A_ exhibited increases in both anxiety and mPFC theta power, while 5-HT_1B_ agonist in the mPFC reduced both anxiety and theta power in the elevated plus maze (Adhikari et al., 2010; Kjaerby et al., 2016)

Impairments of HPC-PFC connectivity have also been associated with depression. Zheng and Zhang (2015) investigated the effects of LTP induction on the oscillatory coupling between vCA1 and mPFC in the chronic unpredictable stress (CUS) model of depression in rats. Remarkably, they showed that CUS rats showed both a weaker LTP induction and reduced theta phase synchrony. Furthermore, they reported a positive correlation between the two measures, suggesting a relationship between these two estimates of neural connectivity. Jia et al. (2019) investigated the oscillatory and antidepressant effects of electrical LFS or HFS in the mPFC. They showed that CUS promoted a reduction of HPC-PFC beta and gamma powers, but electrical stimulations promoted both antidepressant responses and increases in beta and gamma HPC-PFC coherence. Carreno et al. (2016) used optogenetics and chemogenetics to show that the selective activation of the vHPC-mPFC ascending pathway also produces an antidepressant-like response comparable to that observed with sub-anesthetic doses of ketamine. Finally, Marques et al. (2019) investigated iHPC-PFC network dynamics during exposure to controllable or uncontrollable stress. They identified a pattern of increased HPC-PFC theta connectivity that accurately predicted resistant animals but was absent in helpless subjects, suggesting a role of HPC-PFC communication in adaptive stress coping.

### Alzheimer’s Disease

Recent studies in animal models for Alzheimer’s disease (AD) have provided further evidence on HPC-mPFC disruption. Optogenetic activation of hippocampal cells involved in memory formation resulted in long-term memory retrieval in a genetic model of AD, suggesting that retrieval, rather than storage, is impaired in the disease (Roy et al., 2016). Specifically in the HPC-mPFC, alterations in proteins related to AD, such as the decay of calcium-independent phospholipase A2 (iPLA2) and the increase of amyloid-β peptide (Aβ), decreased prefrontal gamma oscillation engagement during spatial working memory (Liu et al., 2016) and abolished CA1-mPFC LTP induction related to spatial working memory deficits, respectively (Shalini et al., 2014). In addition, Bazzigaluppi et al. (2018) showed that the TgF344 genetic rat model of AD, presents lower HPC-mPFC low-gamma power, decreased theta-gamma PAC, and weaker gamma coherence compared to non-transgenic rats. The same frequency bands were analyzed during a stimuli association task performed by adult rats overexpressing the tau protein in the entorhinal cortex (Tanninen et al., 2017). In this case, tau-expressing rats showed attenuated theta-gamma phase-phase and amplitude-amplitude coupling between dHPC and mPFC. Interestingly, these oscillatory activities are also cholinergically modulated, which is impaired in AD (Ballinger et al., 2016; Ferreira-Vieira et al., 2016; Al-Onaizi et al., 2017). In fact, recent evidence has contributed to the advance of new AD treatment through cholinergic neuromodulation. Esteves et al. (2017) showed that chronic nicotine treatment prevented novel object recognition memory deficits and disruption of iHPC-mPFC LTP in the streptozotocin animal model of AD. However, further research is necessary to evaluate the effects of cholinergic neuromodulation on functional connectivity in HPC-mPFC in AD.

## Concluding Remarks

Acetylcholine plays a key role in regulating brain-wide state transition (Huang and Hsu, 2010). The tonic cholinergic drive is fundamental to cortical change from a deactivated to an activated state, allowing HPC-PFC theta synchrony as a form of hippocampal inputs to influence prefrontal activity in time (Teles-Grilo Ruivo et al., 2017). Interestingly, *in vivo* experiments show that cholinergic activation modulates synaptic plasticity bidirectionally, potentiating LTP and facilitating LTD (Lopes Aguiar et al., 2008; Lopes-Aguiar et al., 2013). Also, experimental evidence suggests that nAChR activation can facilitate both forms of synaptic plasticity (α_7_ facilitates LTP and α4β2 LTD) (Sabec et al., 2018). Taken together, these results indicate that ACh enables a specific type of communication to the HPC-PFC pathway (measured as theta rhythm). Once this interaction is established (or because this interaction is established), the cholinergic drive can facilitate activity-dependent synaptic strengthening or weakening. The occurrence of this bidirectional modulation is thought to be essential to regulate excitatory/inhibitory balance and learning.

It has been well established that monoamines provide a fine-tuning functional regulation in HPC-mPFC communication. Through their main signaling pathways in the mPFC, catecholamines (DA and NA) are positive modulators of LTP, while 5-HT is negative (Gurden et al., 2000; Ohashi et al., 2003; Lim et al., 2010). However, D_1_-mediated modulation occurs in an inverted U-shaped dose dependence (Gurden et al., 2000), NA indirectly exerts detrimental effects via amygdala (Lim et al., 2017), and chronic treatment with SSRI can reverse negative impacts of stress (Ohashi et al., 2002; Rocher et al., 2004). Remarkably, we found a qualitative relationship between monoamine modulation of HPC-mPFC synaptic plasticity and network synchrony. Pharmacological manipulations that increase LTP also increase LFP coupling (e.g., Gurden et al., 1999; Benchenane et al., 2010), while negative modulators of LTP decrease it (Zheng and Zhang, 2015; Xu et al., 2016), particularly in theta oscillations. This finding strengthens a link between distinct scales of functional connectivity.

The eCB system is critical to temporal coordination between HPC and mPFC activity. CB_1_ stimulation abolishes spike timing coordination in both HPC and mPFC, which is related to disruption of slow oscillating in the HPC (theta) and fast oscillations in the mPFC (gamma) (Robbe et al., 2006; Kucewicz et al., 2011). In addition, these impairments decrease the synchronicity in HPC-mPFC, and ultimately, behaviors related to vHPC-mPFC communication, such as working memory (Kucewicz et al., 2011). In turn, chronic CB_1_ stimulation impacts short-term and long-term synaptic plasticity in the vHPC-mPFC pathway by promoting an inhibitory imbalance and probably disrupting the vHPC -mPFC information flow (Cass et al., 2014; Renard et al., 2016).

Although considerable progress in understanding the role of neuromodulator systems in the synaptic plasticity of the HPC-mPFC pathway, there are still many gaps that need further elucidation. Regarding monoamines and eCBs, for example, little is known about *in vivo* LTD. All monoamines present two primary receptor families, which pose functional dichotomies that mediate facilitation or suppression of synaptic efficacy (D_1_ vs. D_2_, β vs. α_2_, and 5HT_1_ vs. 5HT_2_). *In vitro* studies have shown the critical roles of DA and NA in PFC and HPC-mPFC LTD (Marzo et al., 2009, 2010; Goto et al., 2010; Banks et al., 2015). Similarly, eCB neuromodulation induces a robust LTD in mPFC slices (Auclair et al., 2000; Riedel and Davies, 2005). Therefore we hypothesize that monoamines and eCB are also critical for LTD in the intact brain. However, we may also reason that while CB_1_ produces a ubiquitous LTD, monoamines may conflict between synaptic facilitation and suppression, which may manifest itself in an inverted U-shape dose dependence, such as in D_1_ functions. In this sense, we highlight that we still miss basic knowledge of pharmacological modulation of HPC-mPFC LTP. Namely, we still do not know the roles of 5-HT2, many of the nAChR, nor any NA receptors in the mPFC and several components related to the eCB system, such as 2-AG and TRPV_1_.

Also, one crucial question is how the *in vitro* findings can be transposed to *in vivo* preparations. Notably, a well-established *in vitro* form of LTD induced by activation of M_1_ receptors lacks *in vivo* demonstration to our knowledge. Although the reviewed results are not directly comparable (i.e., different drug used and experimental design), it does not appear that the *in vivo* effects of muscarinic activation on synaptic efficacy follow the same magnitude and dynamics as the *in vitro* response. Indeed, despite the optimal spatial resolution and control of confounding factors, *in vitro* preparation lacks the intact circuitry and network response that can modulate the interaction in specific circuits. These inputs can include brainstem and forebrain neuromodulatory pathways that produce a physiological complex synergy that allows cognitive and emotional processing. In this respect, most studies investigating monoamine and cholinergic modulation of LFPs were performed in rodents during spontaneous behavioral states or anesthesia. It is reasonable to consider that the HPC-PFC network may present more distinctive patterns of activity and modulation under specific situations such as cognitive demands, motivational states during goal-directed behaviors, or adaptive vs. maladaptive stress coping strategies. These issues should be investigated in the future.

### Future Directions

The concept that neuropsychiatric symptoms could emerge from alterations in information processing within neural networks rather than an imbalance of a specific neurotransmitter is now consolidated (Castrén, 2005). In this view, a promising approach is to investigate the effects of neuropsychiatric drugs on specific networks of the brain. Relevant clinical drugs with non-selective actions make it hard to interpret mechanisms, but investigating their effects in cognitive circuits could be exceptionally informative about the therapeutic effect and elucidate the underlying network functioning that can compensate for the cognitive symptoms. For instance, there is evidence that the different systems may interact to promote efficient modulation of synaptic function (Meunier et al., 2017). Indeed, all monoamines are known to interact between themselves (González-Burgos and Feria-Velasco, 2008; Xing et al., 2016) and with both eCB (Cohen et al., 2019) and cholinergic systems (Lester et al., 2010). However, there is still no knowledge on how these interactions may influence HPC-PFC synapses. In this review, we focused on the basic mechanisms of modulation. However, there is significant evidence that some manipulations only show effects when counteracting the impacts of stress or developmental deficits (Dupin et al., 2006; Goto and Grace, 2006), suggesting a more general role in regulating synaptic homeostasis rather than increasing or decreasing connectivity.

We found a profound difference in research interest on the modulations of the intermediate against the ventral hippocampal inputs to the PFC. While monoamines and cannabinoids were only studied in vHPC inputs, cholinergic modulation was mainly investigated in iHPC. Although evidence has accumulated for a functional distinction across the septotemporal axis of the HPC, we still do not know if the ventral and intermediate HPC inputs into the mPFC are differently modulated by the systems addressed here. Interestingly, most modulation transmitters are released into the PFC in a non-synaptic manner, suggesting a convergence of these modulations onto both inputs. However, the coincident phasic activation of brainstem efferent systems could modulate each input differently across time. For instance, vHPC stimulation markedly increases VTA activity and DA release (Lisman and Grace, 2005). However, iHPC tetanic stimulation was shown to decrease monoamine levels (Lopes Aguiar et al., 2008), suggesting distinct influences of the HPC inputs over the neuromodulatory systems.

One important aspect to recognize is that most studies adopted an artificial stimulation protocol to probe synaptic plasticity. Such robust protocols elicit synchronous spike activity from a large number of neurons in a prolonged and high-frequency manner that unlikely replicate what happens in a physiological state (Bocchio et al., 2017). Thus, it is essential to understand the spontaneous network electrophysiological activities related to synaptic plasticity. Understanding the network effects of synaptic plasticity in the LFP measures while controlling for the artificial stimulation could be useful to understand the dynamics of HPC-mPFC interaction during behavior. It is postulated that oscillatory synchrony facilitates neural communication creating a temporal window in which one region could influence or coordinate the activity in another distant brain region (Fries, 2015). Hence, it is theorized that an increase in synaptic influence would also generate a stronger LFP coupling. Zheng and Zhang (2015) showed that LTP induction in the vHPC-mPFC pathway increased theta phase coupling rather than the power of either region. Also, stimulation protocols that resemble more physiological activity, such as TBS, induce theta-gamma cross-frequency coupling and interfere with memory consolidation (Radiske et al., 2020). Future studies need to investigate the relationship between synaptic plasticity and network coupling in freely moving animals and control for the effects of stimulation *per se*. Furthermore, the effects of other forms of synaptic plasticity in HPC-mPFC network coupling, such as LTD and PPF, remain to be investigated.

## Author Contributions

All authors listed have made a substantial, direct and intellectual contribution to the work, and approved it for publication.

## Conflict of Interest

The authors declare that the research was conducted in the absence of any commercial or financial relationships that could be construed as a potential conflict of interest.

## Publisher’s Note

All claims expressed in this article are solely those of the authors and do not necessarily represent those of their affiliated organizations, or those of the publisher, the editors and the reviewers. Any product that may be evaluated in this article, or claim that may be made by its manufacturer, is not guaranteed or endorsed by the publisher.
